# Modeling the effects of atmospheric pressure on suicide rates in the USA using geographically weighted regression

**DOI:** 10.1371/journal.pone.0206992

**Published:** 2018-12-05

**Authors:** Aaron M. Frutos, Chantel D. Sloan, Ray M. Merrill

**Affiliations:** Department of Public Health, College of Life Sciences, Brigham Young University, Provo, Utah, United States of America; Universita degli Studi Europea di Roma, ITALY

## Abstract

Low atmospheric pressure may increase depression and suicide through inducing hypoxia. Previous studies have not evaluated the geographic variation of this relationship across the United States. Analyses were based on three groupings of age-adjusted completed suicide rates (all suicide, firearm-related suicide, non-firearm-related suicide) from 2286 counties in the United States. Multiple regression was used to determine the overall relationship between atmospheric pressure and completed suicide rates. Geographically weighted regression (GWR) models were used to obtain local coefficient estimates. A negative correlation between atmospheric pressure and completed suicide rates was observed for all three suicide groupings (p*-*value <0.0001). Significant, negative GWR coefficient estimates were located in the West and Northeast for the all suicides and firearm-related suicides, and in the Midwest for non-firearm-related suicides.

## Introduction

In 2014, suicides accounted for 42,773 total deaths and was the second leading cause of death among individuals between 10 and 34 years of age in the United States [1]. Risk factors for suicide include depression, economic and socioeconomic characteristics (e.g., age, race, poverty, etc.), and geographic and environmental factors (e.g. altitude, population density, rainfall, temperature, etc.) [2–20]. In 1997, the Centers for Disease Control and Prevention (CDC) published an article showing that the West census region of the United States had the highest age-adjusted completed suicide rates [19]. Researchers have since examined the hypothesis that geographic factors, such as altitude, may account for this geospatial inequality. Previous research has shown that some, but not all, of the association between altitude and suicide can be explained by selected demographics, population density, and gun ownership [6–8, 10, 11, 13–15].

As altitude increases, atmospheric pressure exponentially decreases [21]. This decline in atmospheric pressure also decreases the partial pressure of inspired oxygen, meaning that less oxygen is absorbed into the body, thereby causing hypoxia [22]. Research suggests that metabolic stress from hypoxia negatively affects mood and increases the risk of depressive symptoms. In turn, depressive symptoms caused by hypoxia may compound with personal stressors and individual suicide characteristics to increase suicides [7, 9, 10, 13–15, 19, 23–28]. In 2015, researchers performed an experiment to determine the effects of hypobaric hypoxia on depression in rats. The study showed that by inducing hypobaric hypoxia through varying air pressures, female rats at the lowest simulated pressures displayed depression-like symptoms [25]. Although there are a number of physiological changes that occur as the body adapts to low atmospheric pressure, one study indicated that mood disorders may disrupt the body’s ability to cope with mild hypoxic conditions. Those who have mood disorders, therefore, may be more likely to commit suicide because of depressive-like symptoms stemming from hypoxia [15].

Previous studies have estimated the effect of altitude, but not atmospheric pressure on completed suicide rates [5–11, 13–15, 18, 19]. Although altitude and atmospheric pressure are directly related, their association is not linear. Additionally, temperature differentially impacts atmospheric pressure, based on altitude (smaller impact at lower altitudes) [21, 29]. In short, the use of altitude and temperature may be a better estimation of the effect of atmospheric pressure on hypoxia than the use of altitude alone.

Another limitation of previous studies is that they did not evaluate the geographic variation of the association between altitude and completed suicide rates [5–11, 14, 15, 18, 19]. By not considering this variation, there is an inherent assumption that the relationship between socio-demographics, mental health, and altitude with suicide is consistent across all locations. This is particularly important because two studies found that the hypothesized relationship between altitude and depression did not exist among older adults in the Himalayas and the Andes [30, 31].

Geographically weighted regression (GWR) is a technique that is specifically designed to test the assumption that associations between the dependent and independent variables remain constant over space [32]. Only one previous study used GWR to describe completed suicide rates in the United States, focusing on indicators of social isolation for males (e.g. marital status, migration status, and unemployment) [20]. The purpose of the current study was to evaluate the relationship between atmospheric pressure and completed suicide rates while accounting for potential geographic variability.

## Materials and methods

### Data sources and preparation

Analyses were based on 1999−2010 county-level age-adjusted suicide rates from counties or county equivalents (parishes, independent cities, etc.) across the contiguous United States. Age-adjusted suicide rates were available from the National Center for Health Statistics (NCHS) through the CDC WONDER database [33]. Mortality rates were obtained from the NCHS annual detailed mortality files, which included resident death certificates, and were age-adjusted using the US 2000 standard population [34].

Deaths were coded according to the 10^th^ revision of the International Statistical Classification of Diseases and Related Health Problems (ICD-10). Suicide deaths were identified using the CDC’s External Cause of Injury Mortality Matrix, which codes each death based on intent and mechanism. Suicide attempts are not reflected in the current study. Suicides were categorized into three groups: all suicide, firearm-related suicide, and non-firearm-related suicide. Suicide was classified as firearm-related and non-firearm-related for two reasons: first, firearm-related suicides accounted for over 50% of all completed suicides during the study period and second, in order to evaluate suicides independent of the potential confounding association between high altitude, gun ownership, and firearm suicides [7, 15, 19].

Because of discontinuities in cause-of-death comparisons for selected diseases between ICD-9 and ICD-10 codes, analysis was focused after 1998. Counties with fewer than 10 deaths were suppressed and not publically available. Counties were marked “unreliable” when the death count was between 11−20 [33]. Suppressed and unreliable death counts were excluded from this study, leaving 2,286 counties (73.6%) for the all suicide group, 1,912 counties (61.5%) for the firearm suicide group and 1,289 counties (41.5%) for the non-firearm suicide group.

Average county-level sex, race, and ethnicity percentages were calculated from Bridged-Race Population Estimates produced by the US Census Bureau and the NCHS, available through CDC Wonder [33]. Average county population density was calculated using the county population from 1999−2010 from the Bridged-Race Population Estimates [33], and county area (in km^2^) available from the US Census Bureau [35]. Average percent below the poverty line for 1999−2010 was calculated from yearly estimates from the US Census Bureau [36]. Average county obesity prevalence for 2004−2005 was obtained from the CDC [37]. Average age-adjusted county cigarette smoking prevalence estimates for 1999−2010 were obtained from data made publically available by Dwyer-Lindgren et al., who used data from the Behavioral Risk Factor Surveillance System (BRFSS) [38]. Percent obese and percent current smokers were included in the model because of their potential to decrease oxygenation in the body, similar to the results of hypoxia in the body [15, 39, 40]. Previous studies recommended the inclusion of obesity in future studies examining the relationship between altitude and suicide [7]. Gun ownership was not included in this study because of the unavailability of accurate county-level data.

Average county-level daily sunlight, and average maximum air temperature from 1999−2010 were obtained through the North America Land Data Assimilation System, available through the CDC Wonder database [33]. Sunlight was included in the model as a potential confounding variable because at higher altitude there is more ultraviolet radiation from the sun [41], and because of the previously observed correlation between lower sunlight and suicide [42].

We calculated population-weighted atmospheric pressure estimates in order to account for locations where high mountains may be present, but most people live in valleys. County-level weighted average altitude values were calculated using information from the United States Geological Survey’s National Elevation Dataset program’s (NED) 1 arc-second dataset made available through ArcGIS Online [43]. This database is composed of 30-meter elevation raster information that covers the entire contiguous United States. We first obtained census tract and county-level shapefiles from the US Census Bureau [44]. Next, we aggregated the altitude values from the NED 1-arc-second data set to the census tract (neighborhood) level. Next, using population values from the 2000 U.S. census for each tract, we calculated county population-weighted altitude. Then, using the hypsometric formula for atmospheric pressure, we calculated county-level atmospheric pressure estimates with county-level weighted altitude, and county average maximum temperature values [29].

### Data analysis

Data were matched by county FIPS codes and then joined to shapefiles and displayed in ArcGIS Pro (v 2.0.1). The following county-level variables were used in the study models: atmospheric pressure, average daily sunlight, percent male, percent Hispanic, percent Caucasian, percent below the poverty line, population density, percent current smokers, and percent obese. Ordinary least squares regression (OLS) was conducted and Moran’s I, a cluster detection measure, was subsequently applied to measure spatial autocorrelation in model residuals. Spatial autocorrelation was significantly positive (p-value <0.0001), indicating clustering of model residuals. This clustering indicates that the association between the independent and dependent variables may vary by region.

Because of spatial autocorrelation in the global model, GWR was selected as an analytical method to determine local variation in model coefficients. GWR uses a circular bandwidth that moves across the geographic area, calculating new regression models within the bandwidth. Bandwidth size can be either geographically stable (always have the same diameter) or population-based (size of the bandwidth varies to always include a minimum number of data points). For this study, we selected a population-based model.

The optimal bandwidth size was determined by comparing Akaike information criterion corrected (AICc), a measure of model fit, of GWR models with different size bandwidths. The bandwidth size that produced the model with the lowest AICc value was determined to be the bandwidth size that produced the best fitting model and, therefore, was the most stable for the data. Because of differences in the number of available county data for suicide mortality by method, separate bandwidth sizes were used for each suicide category model (215 for all suicide, 243 for firearm suicide, and 108 for non-firearm suicide). Bonferroni adjusted p-values were used to account for multiple testing and to determine statistically significant beta coefficients. Model variables were summarized and collinearity between variables was checked using SAS 9.4 (SAS Institute, Cary, NC, USA, 2012).

GWR model diagnostics were compared with those from OLS regression. GWR ANOVA is a method that compares the sums of squares from OLS and GWR models. GWR results were a better fit for each suicide (all, firearm, no firearm) model based on statistically significant GWR ANOVA F-tests with p-values less than 0.0001. We used the software package GWR v. 4.09, developed at the National Centre for Geocomputation, to conduct GWR analysis as well as global OLS assessment of the data [45]. ArcGIS Pro was used to conduct Moran’s I. Analyses were conducted separately for each suicide method category.

Separate models were created using weighted altitude or log-transformed weighted altitude instead of atmospheric pressure. AICc were generally lower for each of the six models (OLS and GWR for each of the three suicide groupings) using atmospheric pressure rather than weighted or log-transformed weighted altitude. This indicates that atmospheric pressure produced a better fitting mode. Therefore, we retained atmospheric pressure in our final models rather than switching it for weighted altitude or log-transformed weighted altitude.

## Results

### Population demographics

**Fig 1:**
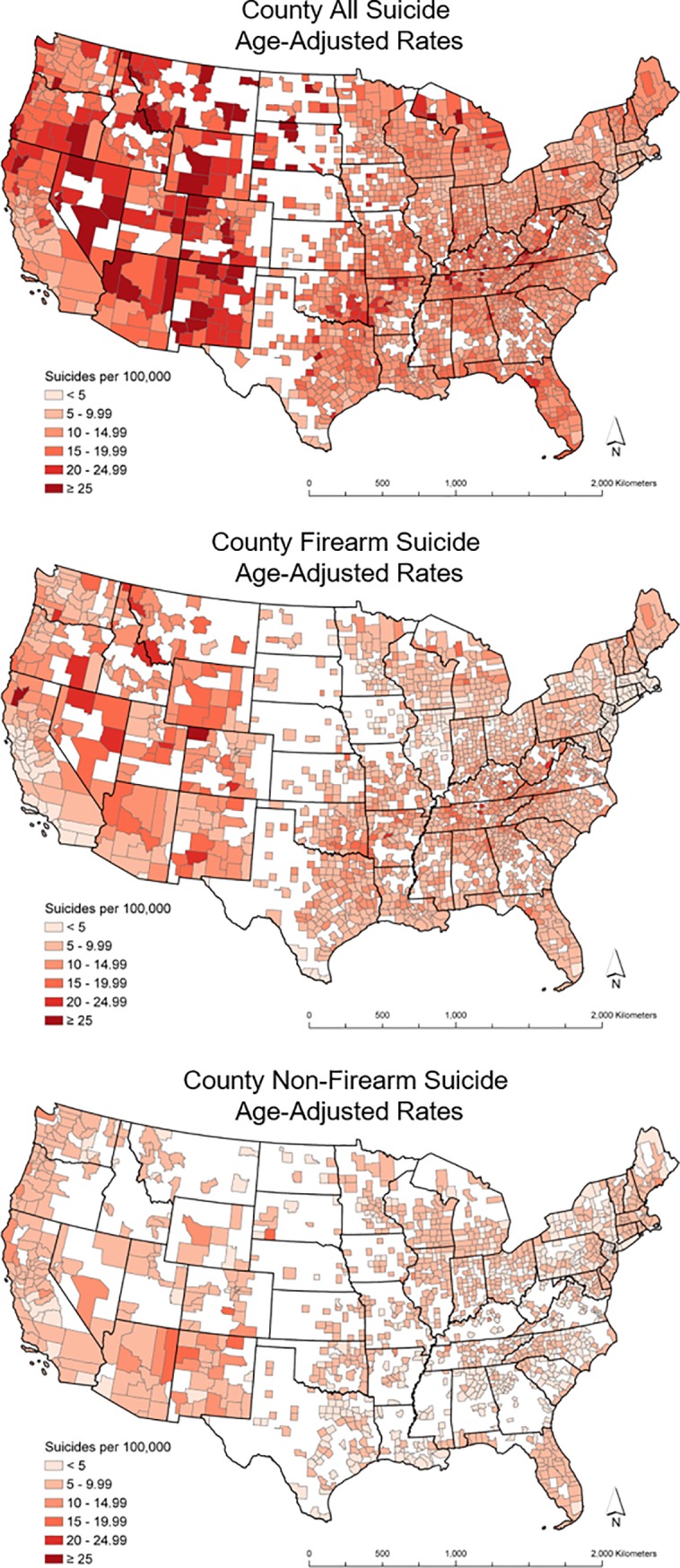
Country suicide rates. The shapefiles used to make this figure are from the US Census Bureau and are therefore reproducible by law.

Age-adjusted suicide rates for each suicide grouping, and geographic and demographic summary statistics for all 2,286 counties are presented in Table 1. The West census region of the United States had higher suicide rates than other regions for all, firearm, and non-firearm suicides (Fig 1). Mean firearm suicide rates were higher than non-firearm suicide rates in most counties (see S1 Table to compare specific method of suicide across the contiguous United Sates). The West census region also had lower levels of atmospheric pressure and a higher percentage of Hispanics. The South census region of the US had a higher county percent of current smokers and percent obese and lower county percent of Caucasians. Across the United States, the average amount of daily sunlight decreased at higher latitudes. The variables considered had low multicollinearity (variation inflation factors (VIF) < 2.9).

**Table 1:** County summary statistics.

**Variable**		**N**	**Mean**	**SD**	**Median**	**Minimum**	**Maximum**
**Atmospheric Pressure (hPa)**		2286	973.41	47.30	985.42	690.00	1014.34
**Weighted Altitude (m)**		2286	354.02	447.40	236.31	-9.53	3246.61
**Daily Average Max Air Temp (°C)**		2286	18.38	4.86	18.18	5.16	30.74
**Sun (KJ/m^2^)**		2286	16185.06	1514.24	15989.48	12998.22	20942.67
**Male (%)**		2286	49.54	1.64	49.30	44.96	64.87
**Hispanic (%)**		2286	6.91	11.39	2.76	0.36	96.64
**Caucasian (%)**		2286	87.20	14.71	93.34	3.87	99.65
**Poverty (%)**		2286	14.31	5.23	13.81	2.98	41.68
**Population Density (People per km^2^)**		2286	126.46	759.50	26.06	0.28	26357.47
**Current Smoker (%)**		2286	26.25	3.90	26.58	9.30	39.74
**Obese (%)**		2286	25.57	3.34	25.81	12.05	37.62
**Variable**		**N**	**Mean**	**SD**	**Median**	**Minimum**	**Maximum**
All Suicide*		2286	13.85	4.59	13.05	4.28	57.20
Suicide by Firearm*	1912		8.61	3.55	8.20	0.77	25.60
Suicide excluding Firearms*	1289		5.80	2.67	5.49	1.00	50.94

*Age Adjusted Rate per 100,000 (US 2000 Standard Population)

### OLS model results

Atmospheric pressure was negatively related to age-adjusted suicide mortality rates in all three categories of suicide *(*p-value *<*0.0001), after adjusting for demographic variables and daily average sunlight exposure (Table 2). The estimated coefficients for atmospheric pressure (hPa) for firearm suicide are almost three times the estimated coefficients for non-firearm suicide (-0.035 compared with -0.013). The adjusted R^2^ value for the non-firearm suicide model was much lower than the firearm suicide model (0.14 compared to 0.53).

**Table 2:** OLS model summaries.

**All Suicide (n = 2286)**			
**Adjusted R^2^**	0.43		
**Variable**	**Estimate**	**95% CI**	**p-value**
**Atmospheric Pressure (hPa)**	-0.049	(-0.053, -0.046)	<0.0001
**Sun (KJ/m^2^)**	0.001	(0.000, 0.001)	<0.0001
**Male (%)**	0.053	(-0.038, 0.144)	0.2548
**Hispanic (%)**	-0.072	(-0.089, -0.055)	<0.0001
**Caucasian (%)**	0.033	(0.019, 0.047)	<0.0001
**Poverty (%)**	0.135	(0.093, 0.178)	<0.0001
**Population Density (People per km^2^)**	-0.000	(-0.000, -0.000)	0.0247
**Current Smoker (%)**	0.399	(0.341, 0.456)	<0.0001
**Obese (%)**	-0.118	(-0.189, -0.047)	0.0012
**Firearm Suicide (n = 1912)**			
**Adjusted R^2^**	0.53		
**Variable**	**Estimate**	**95% CI**	**p-value**
**Atmospheric Pressure (hPa)**	-0.035	-0.037, -0.032	<0.0001
**Sun (KJ/m^2^)**	0.001	0.001, 0.001	<0.0001
**Male (%)**	0.080	0.005, 0.156	0.0378
**Hispanic (%)**	-0.099	-0.112, -0.085	<0.0001
**Caucasian (%)**	0.073	0.062, 0.084	<0.0001
**Poverty (%)**	0.166	0.134, 0.199	<0.0001
**Population Density (People per km^2^)**	-0.000	-0.000, -0.000	0.0382
**Current Smoker (%)**	0.225	0.181, 0.269	<0.0001
**Obese (%)**	0.088	0.034, 0.141	0.0014
**Non-Firearm Suicide (n = 1289)**			
**Adjusted R^2^**	0.14		
**Variable**	**Estimate**	**95% CI**	**p-value**
**Atmospheric Pressure (hPa)**	-0.013	-0.017, -0.010	<0.0001
**Sun (KJ/m^2^)**	-0.000	-0.000, 0.000	0.0888
**Male (%)**	0.166	0.066, 0.267	0.0012
**Hispanic (%)**	0.002	-0.015, 0.019	0.8167
**Caucasian (%)**	-0.021	-0.035, -0.008	0.0020
**Poverty (%)**	0.064	0.020, 0.107	0.0045
**Population Density (People per km^2^)**	-0.000	-0.000, 0.000	0.3348
**Current Smoker (%)**	0.159	0.106, 0.212	<0.0001
**Obese (%)**	-0.153	-0.218, -0.088	<0.0001

### GWR model results

GWR model summaries are provided in Table 3. For each suicide grouping, GWR ANOVA indicated that GWR produced better fitting models (p-value < 0.0001). Additionally, adjusted R^2^ values increased for all models (0.43 to 0.63 for all suicides, 0.53 to 0.66 for firearm suicide, and 0.14 to 0.52 for non-firearm suicide).

**Table 3:** GWR model summaries.

**All Suicide (n = 2286)**					
**Adjusted R^2^**	0.63				
**Variable**	**Mean**	**SD**	**Median**	**Min**	**Max**
**Atmospheric Pressure (hPa)**	-0.027	0.045	-0.031	-0.190	0.141
**Sun (KJ/m^2^)**	0.001	0.001	0.001	-0.002	0.006
**Male (%)**	-0.021	0.229	-0.017	-0.800	0.935
**Hispanic (%)**	-0.124	0.091	-0.118	-0.543	0.170
**Caucasian (%)**	0.069	0.061	0.079	-0.270	0.187
**Poverty (%)**	0.111	0.120	0.093	-0.149	0.599
**Population Density (People per km^2^)**	0.001	0.002	0.000	-0.011	0.014
**Current Smoker (%)**	0.416	0.198	0.407	-0.058	0.992
**Obese (%)**	-0.043	0.203	-0.025	-0.962	0.615
**Firearm Suicide (n = 1912)**					
**Adjusted R^2^**	0.66				
**Variable**	**Mean**	**SD**	**Median**	**Min**	**Max**
**Atmospheric Pressure (hPa)**	-0.035	0.035	-0.035	-0.116	0.075
**Sun (KJ/m^2^)**	0.001	0.001	0.000	-0.001	0.005
**Male (%)**	0.087	0.189	0.090	-0.349	0.556
**Hispanic (%)**	-0.102	0.063	-0.096	-0.335	0.152
**Caucasian (%)**	0.079	0.039	0.078	-0.017	0.194
**Poverty (%)**	0.107	0.085	0.107	-0.062	0.387
**Population Density (People per km^2^)**	-0.001	0.002	-0.000	-0.008	0.003
**Current Smoker (%)**	0.281	0.135	0.271	-0.016	0.565
**Obese (%)**	0.063	0.113	0.070	-0.340	0.521
**Non-Firearm Suicide (n = 1289)**					
**Adjusted R^2^**	0.52				
**Variable**	**Mean**	**SD**	**Median**	**Min**	**Max**
**Atmospheric Pressure (hPa)**	-0.001	0.023	-0.003	-0.129	0.071
**Sun (KJ/m^2^)**	0.000	0.001	0.000	-0.002	0.003
**Male (%)**	-0.008	0.438	-0.080	-1.398	3.920
**Hispanic (%)**	-0.013	0.065	-0.013	-0.319	0.375
**Caucasian (%)**	0.018	0.047	0.026	-0.135	0.160
**Poverty (%)**	0.033	0.107	0.027	-0.260	0.667
**Population Density (People per km^2^)**	0.001	0.001	0.001	-0.004	0.009
**Current Smoker (%)**	0.134	0.227	0.156	-2.121	0.439
**Obese (%)**	-0.056	0.247	-0.100	-0.469	2.435

**Fig 2:**
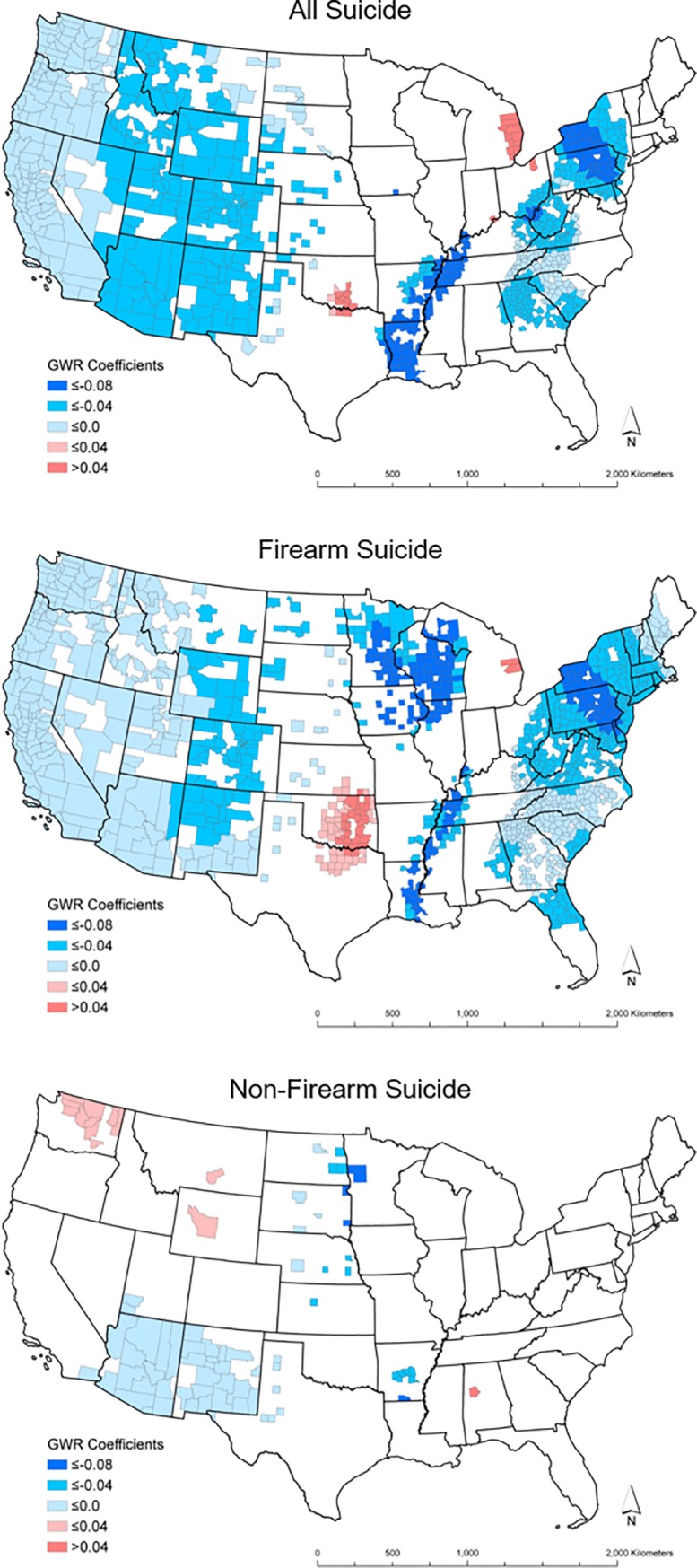
GWR uncorrected significant atmospheric pressure coefficients. The shapefiles used to make this figure are from the US Census Bureau and are therefore reproducible by law.

**Fig 3:**
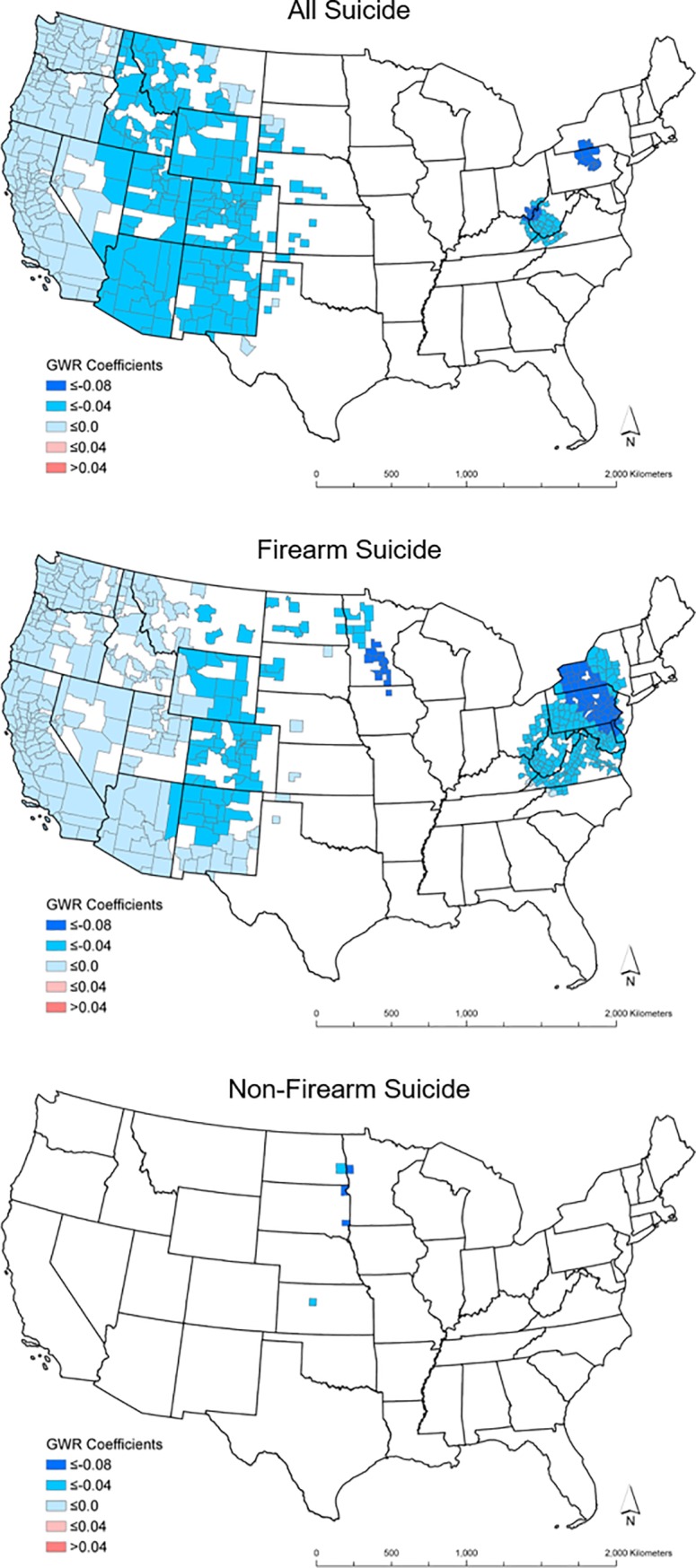
GWR Bonferroni corrected significant atmospheric pressure coefficients. The shapefiles used to make this figure are from the US Census Bureau and are therefore reproducible by law.

Summary statistics for statistically significant and Bonferroni corrected significant atmospheric pressure coefficients (p-value <0.05) are displayed (Tables 4, 5 respectively), and significant and Bonferroni corrected significant coefficients are mapped in Figs 2 and 3. A total of 901 counties in the all suicide, 1242 counties in the firearm suicide, and 81 counties in the non-firearm suicide models had statistically significant atmospheric pressure coefficients. After the Bonferroni correction, 385 counties in the all suicide, 574 counties in the firearm suicide, and 5 counties in the non-firearm suicide models had significant atmospheric pressure coefficients.

**Table 4:** Summary of significant uncorrected GWR atmospheric pressure coefficients.

Variable	N	Mean	SD	Median	Minimum	Maximum
**All Suicide**	901	-0.056	0.040	-0.052	-0.190	0.141
**Suicide by Firearm**	1242	-0.049	0.030	-0.045	-0.116	0.070
**Non-Firearm Suicide**	81	-0.027	0.033	-0.019	-0.129	0.065

**Table 5:** Summary of significant Bonferroni corrected GWR atmospheric pressure coefficients.

Variable	N	Mean	SD	Median	Minimum	Maximum
**All Suicide**	385	-0.051	0.018	-0.049	-0.110	-0.029
**Suicide by Firearm**	574	-0.053	0.020	-0.049	-0.099	-0.021
**Non-Firearm Suicide**	5	-0.091	0.030	-0.096	-0.129	-0.048

Statistically significant negative coefficients are consistently present in the West census region for both the all suicide and firearm suicide groupings, even after the Bonferroni correction. Parts of the eastern United States remained significant after the Bonferroni correction, primarily in New York, Pennsylvania, and West Virginia. In the non-firearm suicide model, following the correction, there were only five counties with significant negative coefficients and all were located in the Midwest census region. After the Bonferroni correction, there were no significant, positive atmospheric pressure coefficients in the any of the three models. Larger negative coefficients (≤ -0.08) were located in the Northeast census region for the all and firearm suicide models and in the Midwest for the firearm and non-firearm suicide models after the Bonferroni correction.

## Discussion

This study evaluated the relationship between atmospheric pressure and suicide rates, accounting for potential geographic variability. OLS regression showed that atmospheric pressure was globally related to suicide rates for all, firearm, and non-firearm types of suicide, as consistent with previous findings involving altitude (5–11, 13–15, 19). According to the all suicide model, an increase in atmospheric pressure by 100 hPa was associated with a decrease in suicide rates by 4.9 per 100,000, holding all else constant.

The observed global relationship between atmospheric pressure and non-firearm suicide indicates that there is a relationship between suicide and atmospheric pressure independent of the association between atmospheric pressure and the distribution of firearms. The relationship between non-firearm suicide and atmospheric pressure is consistent with previous research [7, 15]. This association in the current study persists after adjusting for daily sunlight exposure, percent male, percent Hispanic, percent Caucasian, percent below the poverty line, population density, percent current smoker percent, and percent obese.

The geographic distribution of significant GWR atmospheric pressure coefficients is similar for the all suicide and firearm suicide models. This similarity is likely because of the larger proportion of suicides committed by firearm compared with other approaches. The West census region and portions of the eastern United States showed a consistent, significant negative association between atmospheric pressure and suicide rates. There were larger negative atmospheric pressure coefficients in the Northeast census region than in West. This is unexpected because greater variation in atmospheric pressure in the West combined with higher suicide rates in the Mountain West should result in larger GWR negative atmospheric pressure coefficients than in the East census region. There was more missing data in the West and neighboring regions of the Midwest than in the Northeast. This missing data is not random (counties reporting less than 20 suicides in the study period) and makes the GWR bandwidth geographically wider and potentially less stable. This may explain the larger negative coefficients in the Northeast compared with the West.

The non-firearm model showed a cluster of uncorrected significantly negative coefficients in the southwestern United States (Arizona and Mexico), while the five corrected significant coefficients were only located in the Midwest. The Mountain West census region, as well as the eastern United States, did not have significant negative coefficients in the non-firearm model. This may be because of the large amount of missing county data, particularly in the Mountain West and Midwest census regions, or unaccounted risk factors like substance abuse that may replace firearms as a means to suicide.

A previous study that showed suffocation was the most common method of suicide for American Indian/Alaskan Native (AIAN) teenagers and young adults [46]. Hence, we added percent AIAN to the non-firearm suicide model. However, after adding this variable to the model there remained a significant negative relationship between atmospheric pressure and non-firearm suicides, even in states like Arizona and New Mexico that have a relatively high percentage of AIANs. More county data in the South, Midwest, and Northwest is needed to ensure that the negative relationship between non-firearm suicide and atmospheric pressure is consistent across the entire United States.

The relationship between atmospheric pressure and suicide depends on the approach taken for committing suicide. The OLS coefficients for firearm suicide were almost three times those for the non-firearm suicide model (-0.035 vs. -0.013). Part of this may be explained by the larger number of suicide attempts and completions with firearms. However, the firearm suicide model did not control for firearm ownership. Previous studies have found a positive correlation between firearm ownership and suicides [47, 48]. However, the distribution of firearms does not completely explain the relationship between atmospheric pressure and suicide rates. This also underscores the importance of obtaining accurate firearm ownership information to further examine the impact of firearms on suicide rates in the United States.

A primary strength of GWR is the evaluation of regional associations for all data points. While the observed significant negative associations between atmospheric pressure and suicide in the Mountain West census region and nearby counties were expected, GWR showed that the same relationship existed for counties in the East. This indicates that for both all suicides and firearm-related suicides, low atmospheric pressure was also a risk factor for suicides on the East Coast. In other words, the observed relationship between atmospheric pressure and suicide was not exclusive to the Mountain West region. Counties with significant atmospheric coefficient estimates have varying suicide rates, demographics, and atmospheric pressures.

The decision to use estimated atmospheric pressure instead of weighted altitude was because atmospheric pressure, not altitude, is directly responsible for the hypoxic conditions that may be the neurobiological basis of the association between suicide and altitude. Temperature does have an impact on atmospheric pressure, but is mitigated by the low number of high-altitude counties; if more high altitude counties with varying average maximum temperatures were included, the impact of temperature on atmospheric pressure would likely be greater.

While considering the differences between weighted altitude and atmospheric pressure, we found that the correlation coefficient between atmospheric pressure and weighted altitude was 0.998. This indicates that the differences between the use of atmospheric pressure or weighted altitude are small. Global regression results were generally better fitting when considering atmospheric pressure instead of weighted altitude or log-transformed weighted altitude. Although the use atmospheric pressure instead of altitude may not have led to different conclusions, conceptually, atmospheric pressure is a more appropriate measure.

A limitation of past studies was that elevation was estimated using simple average altitude values or altitude point estimates from state capitals or government offices [5–11, 13–15, 18, 19]. This does not account for the altitude where residents within those counties reside. Mean altitudes involving large mountain ranges overestimate the average altitude of a county or state relative to where the people live. This overestimation may bias the estimated influence of altitude on suicide. Hence, the current study uses population-based weighted county-level estimates of altitude.

Conclusions about atmospheric pressure do not address seasonal changes in pressure. Based on previous research, we do not expect suicide to be as dependent on seasonal fluctuation in pressure as on perpetual exposure to high- or low-pressure environments [7, 9, 10, 13–15, 19, 23–28]. As previously mentioned, firearm ownership data was not available at the county-level, which may influence the relationship between atmospheric pressure and firearm-related suicides. In addition, suicide rates were not available or considered unreliable for some counties in certain years, limiting the number of counties with usable data. This was addressed by aggregating data over selected years.

## Conclusion

Previous studies have evaluated the association between altitude and suicide rates. The current study evaluated the association between atmospheric pressure (a function of both altitude and temperature) and suicide rates. Atmospheric pressure may be more directly associated with hypoxia and its corresponding influence on depression and suicide. The association between atmospheric pressure and suicide rates persisted after adjusting for selected demographic variables and sunlight exposure. This was observed globally and locally for all, firearm, and non-firearm suicides. The relationship was consistent across a variety of geographic areas with different characteristic and altitudes.

## Supporting information

S1 Table 1999–2010 main suicide methods in the contiguous United States. (DOCX)

S2 Table Demographic of counties with significant uncorrected, negative atmospheric pressure coefficients. (DOCX)

S3 Table Demographic of counties with significant Bonferroni corrected, negative atmospheric pressure coefficients. (DOCX)

S1 File 1999–2010 GWR suicide, atmospheric pressure, demographic data. (XLSX)

## References

[1] Web-based Injury Statistics Query and Reporting System [Internet]. Centers for Disease Control and Prevention. 2017 [cited 10 April 2017]. Available from: https://www.cdc.gov/injury/wisqars/index.html.

[2] Achté K. Depression and suicide. Psychopathology. 1986;19(2):210–214. 3554303 10.1159/000285157

[3] Berman A. Depression and suicide In: Gotlib I, Hammen C, ed. by. Handbook of Depression. 2nd ed New York, NY: Guilford Press; 2010 p. 708.

[4] Minkoff K, Bergma E, Beck A, Beck R. Hopelessness, depression, and attempted suicide. American Journal of Psychiatry. 1973;130(4):455–9. 10.1176/ajp.130.4.455 4691303 10.1176/ajp.130.4.455

[5] Betz M, Valley M, Lowenstein S, Hedegaard H, Thomas D, Stallones L et al Elevated suicide rates at high altitude: Sociodemographic and health issues may be to blame. Suicide and Life-Threatening Behavior. 2011;41(5):562–573. 10.1111/j.1943-278X.2011.00054.x 21883411 10.1111/j.1943-278X.2011.00054.x

[6] Alameda-Palacios J, Ruiz-Ramos M, García-Robredo B. Suicide mortality in Andalusia, Spain: Geographical distribution and relationship with antidepressants, altitude and socioeconomic inequalities. Revista Española de Salud Pública. 2015;89(3):283–293. 10.4321/S1135-57272015000300006 26388342 10.4321/S1135-57272015000300006

[7] Brenner B, Cheng D, Clark S, Camargo C. Positive association between altitude and suicide in 2584 U.S. counties. High Altitude Medicine & Biology. 2011;12(1):31–35. 21214344 10.1089/ham.2010.1058 PMC3114154

[8] Bezerra Filho J, Werneck G, Almeida R, Oliveira M, Magalhães F. Socio-demographic determinants of suicide in the State of Rio de Janeiro, Brazil, 1998–2002. Cadernos de Saúde Pública. 2012;28(5):833–844. 22641507 10.1590/s0102-311x2012000500003

[9] Cheng D. Higher suicide death rate in Rocky Mountain states and a correlation to altitude. Wilderness & Environmental Medicine. 2010;21(2):177–178. 20591388 10.1016/j.wem.2010.01.004

[10] Haws C, Gray D, Yurgelun-Todd D, Moskos M, Meyer L, Renshaw P. The possible effect of altitude on regional variation in suicide rates. Medical Hypotheses. 2009;73(4):587–590. 10.1016/j.mehy.2009.05.040 19581053 10.1016/j.mehy.2009.05.040

[11] Helbich M, Blüml V, Leitner M, Kapusta N. Does altitude moderate the impact of lithium on suicide? A spatial analysis of Austria. Geospatial health. 2013;7(2):209. 10.4081/gh.2013.81 23733285

[12] Hong J, Knapp M. Geographical inequalities in suicide rates and area deprivation in South Korea. The Journal of Mental Health Policy and Economics. 2013;16(3):109–19. 24327481

[13] Huber R, Coon H, Kim N, Renshaw P, Kondo D. Altitude is a risk factor for completed suicide in bipolar disorder. Medical Hypotheses. 2014;82(3):377–381. 10.1016/j.mehy.2014.01.006 24495565 10.1016/j.mehy.2014.01.006 PMC3981603

[14] Kim J, Choi N, Lee Y, An H, Kim N, Yoon H et al High altitude remains associated with elevated suicide rates after adjusting for socioeconomic status: A study from South Korea. Psychiatry Investigation. 2014;11(4):492 10.4306/pi.2014.11.4.492 25395983 10.4306/pi.2014.11.4.492 PMC4225216

[15] Kim N, Mickelson J, Brenner B, Haws C, Yurgelun-Todd D, Renshaw P. Altitude, gun ownership, rural areas, and suicide. American Journal of Psychiatry. 2011;168(1):49–54. 10.1176/appi.ajp.2010.10020289 20843869 10.1176/appi.ajp.2010.10020289 PMC4643668

[16] Micciolo R, Zimmermann-Tansella C, Williams P, Tansella M. Geographical variation in the seasonality of suicide. Journal of Affective Disorders. 1988;15(2):163–168. 2975687 10.1016/0165-0327(88)90085-7

[17] Miller M, Azrael D, Barber C. Suicide mortality in the United States: The importance of attending to method in understanding population-level disparities in the burden of suicide. Annual Review of Public Health. 2012;33(1):393–408. 10.1146/annurev-publhealth-031811-124636 22224886

[18] Oka M, Kubota T, Tsubaki H, Yamauchi K. Analysis of impact of geographic characteristics on suicide rate and visualization of result with Geographic Information System. Psychiatry and Clinical Neurosciences. 2014;69(6):375–382. 10.1111/pcn.12254 25384900 10.1111/pcn.12254

[19] Regional variations in suicide rates—United States, 1990–1994. The Journal of the American Medical Association. 1997;278(12):974 9307334

[20] Trgovac A, Kedron P, Bagchi-Sen S. Geographic variation in male suicide rates in the United States. Applied Geography. 2015;62:201–209.

[21] Wallace J, Hobbs P. Atmospheric Science: An Introductory Survey. 2nd ed Amsterdam: Elsevier Academy Press; 2006.

[22] Peacock A. Oxygen at high altitude. British Medical Journal. 1988;317(7165):1063–1066.https://www.ncbi.nlm.nih.gov/pmc/articles/PMC1114067/10.1136/bmj.317.7165.1063 PMC1114067 9774298

[23] Gamboa J, Caceda R, Arregui A. Is depression the link between suicide and high altitude?. High Altitude Medicine & Biology. 2011;12(4):403–404. 22206568 10.1089/ham.2011.1014 PMC6463990

[24] DelMastro K, Hellem T, Kim N, Kondo D, Sung Y, Renshaw P. Incidence of major depressive episode correlates with elevation of substate region of residence. Journal of Affective Disorders. 2011;129(1–3):376–379. 10.1016/j.jad.2010.10.001 21074272 10.1016/j.jad.2010.10.001 PMC4638173

[25] Kanekar S, Bogdanova O, Olson P, Sung Y, D'Anci K, Renshaw P. Hypobaric hypoxia induces depression-like behavior in female Sprague-Dawley rats, but not in males. High Altitude Medicine & Biology. 2015;16(1):52–60. 25803141 10.1089/ham.2014.1070 PMC4376288

[26] Li X, Wu X, Fu C, Shen X, Wu Y, Wang T. Effects of acute mild and moderate hypoxia on human mood state. Space Medicine and Medical Engineering. 2000;13(1):1–5. 12212624

[27] Shukitt-Hale B, Banderet L, Lieberman H. Elevation-dependent symptom, mood, and performance changes produced by exposure to hypobaric hypoxia. The International Journal of Aviation Psychology. 1998;8(4):319–334. 10.1207/s15327108ijap0804_1 11542275 10.1207/s15327108ijap0804_1

[28] Young S. Elevated incidence of suicide in people living at altitude, smokers and patients with chronic obstructive pulmonary disease and asthma: Possible role of hypoxia causing decreased serotonin synthesis. Journal of Psychiatry & Neuroscience. 2013;38(6):423–426. 24148847 10.1503/jpn.130002 PMC3819157

[29] Atmospheric pressure from altitude Calculator [Internet]. Keisan Online Calculator. 2017 [cited 10 April 2017]. Available from: http://keisan.casio.com/exec/system/1224579725.

[30] Ishikawa M, Yamanaka G, Yamamoto N, Nakaoka T, Okumiya K, Matsubayashi K et al Depression and altitude: Cross-sectional community-based study among elderly high-altitude residents in the Himalayan regions. Culture, Medicine, and Psychiatry. 2015;40(1):1–11. 10.1007/s11013-015-9462-7 26162459

[31] Ishikawa M, Yamanaka G, Nakajima S, Suwa K, Matsuda A, Nakaoka T et al Association between high altitude and depression in the Himalayas and the Andes. Japanese Journal of Geriatrics. 2013;50(3):330–334. 23979333 10.3143/geriatrics.50.330

[32] Nakaya T, Fotheringham A, Brunsdon C, Charlton M. Geographically weighted Poisson regression for disease association mapping. Statistics in Medicine. 2005;24(17):2695–2717. 10.1002/sim.2129 16118814 10.1002/sim.2129

[33] CDC WONDER [Internet]. Centers for Disease Control and Prevention. 2017 [cited 10 April 2017]. Available from: https://wonder.cdc.gov/.

[34] Compressed Mortality File [Internet]. Centers for Disease Control and Prevention. 2017 [cited 10 April 2017]. Available from: https://www.cdc.gov/nchs/data_access/cmf.htm.

[35] American FactFinder [Internet]. United States Census Bureau. 2017 [cited 12 April 2017]. Available from: https://factfinder.census.gov/faces/nav/jsf/pages/index.xhtml.

[36] Model-based Small Area Income & Poverty Estimates (SAIPE) for School Districts, Counties, and States [Internet]. United States Census Bureau. 2017 [cited 10 April 2017]. Available from: https://www.census.gov/did/www/saipe/.

[37] County Data Indicators [Internet]. Centers for Disease Control and Prevention. 2017 [cited 10 April 2017]. Available from: https://www.cdc.gov/diabetes/data/countydata/countydataindicators.html.

[38] Dwyer-Lindgren L, Mokdad A, Srebotnjak T, Flaxman A, Hansen G, Murray C. Cigarette smoking prevalence in US counties: 1996–2012. Population Health Metrics. 2014;12(1). 10.1186/1478-7954-12-5 PMC3987818 24661401

[39] Aubin H, Berlin I, Reynaud M. Current smoking, hypoxia, and suicide. American Journal of Psychiatry. 2011;168(3):326–327. 10.1176/appi.ajp.2010.10101501 21368309 10.1176/appi.ajp.2010.10101501

[40] Littleton S, Tulaimat A. The effects of obesity on lung volumes and oxygenation. Respiratory Medicine. 2017;124:15–20. 10.1016/j.rmed.2017.01.004 28284316 10.1016/j.rmed.2017.01.004

[41] Blumthaler M, Webb A, Seckmeyer G, Bais A, Huber M, Mayer B. Simultaneous spectroradiometry: A study of solar UV irradiance at two altitudes. Geophysical Research Letters. 1994;21(25):2805–2808.

[42] Vyssoki B, Kapusta N, Praschak-Rieder N, Dorffner G, Willeit M. Direct effect of sunshine on suicide. JAMA Psychiatry. 2014;71(11):1231 10.1001/jamapsychiatry.2014.1198 25208208 10.1001/jamapsychiatry.2014.1198

[43] Ground Surface Elevation - 30m [Internet]. ArcGIS. 2017 [cited 12 April 2017]. Available from: http://www.arcgis.com/home/item.html?id=0383ba18906149e3bd2a0975a0afdb8e.

[44] TIGER/Line Shapefiles and TIGER/Line Files [Internet]. United States Census Bureau. 2017 [cited 12 April 2017]. Available from: https://www.census.gov/geo/maps-data/data/tiger-line.html.

[45] GWR4 for Windows [Internet]. Geographically Weighted Modelling. 2017 [cited 10 April 2017]. Available from: http://gwr.maynoothuniversity.ie/gwr4-software/.

[46] Jiang C, Mitran A, Miniño A, Ni H. Racial and gender disparities in suicide among young adults aged 18–24: United States. Health E-Stats. 2015.

[47] Miller M, Warren M, Hemenway D, Azrael D. Firearms and suicide in US cities. Injury Prevention. 2013;21(e1):e116–e119. 10.1136/injuryprev-2013-040969 24302479 10.1136/injuryprev-2013-040969

[48] Siegel M, Rothman E. Firearm ownership and suicide rates among US men and women, 1981–2013. American Journal of Public Health. 2016;106(7):1316–1322. 10.2105/AJPH.2016.303182 27196643 10.2105/AJPH.2016.303182 PMC4984734

