# Supplementary material for: Modeling the effects of atmospheric pressure on suicide rates in the USA using geographically weighted regression
Source: PLoS One. 2018 Dec 5;13(12):e0206992. doi: 10.1371/journal.pone.0206992 (PMC6281181; doi:10.1371/journal.pone.0206992)
Supplement: S1 Table — (DOCX) [file pone.0206992.s001.docx]

| **Injury Mechanism** | **Deaths** | **Age Adjusted Rate*** |
| --- | --- | --- |
| **Cut/Pierce** | 6,724 | 0.19 |
| **Drowning** | 4,346 | 0.12 |
| **Fall** | 8,206 | 0.23 |
| **Fire/Flame** | 1,911 | 0.05 |
| **Firearm** | 207,025 | 5.82 |
| **Poisoning** | 68,915 | 1.94 |
| **Suffocation** | 86,591 | 2.47 |

*Age Adjusted Rate per 100,000 (US 2000 Standard Population)
