# Supplementary material for: Modeling the effects of atmospheric pressure on suicide rates in the USA using geographically weighted regression
Source: PLoS One. 2018 Dec 5;13(12):e0206992. doi: 10.1371/journal.pone.0206992 (PMC6281181; doi:10.1371/journal.pone.0206992)
Supplement: S2 Table — (DOCX) [file pone.0206992.s002.docx]

| **Variable** | **N** | **Mean** | **SD** | **Median** | **Minimum** | **Maximum** |
| --- | --- | --- | --- | --- | --- | --- |
| **All Suicide** | 873 | 15.16 | 5.66 | 13.89 | 5.79 | 57.20 |
| **Atmospheric Pressure (hPa)** | 873 | 948.08 | 66.70 | 977.08 | 690.00 | 1014.34 |
| **Weighted Altitude (m)** | 873 | 591.92 | 636.42 | 310.17 | -9.53 | 3246.61 |
| **Daily Average Max Air Temp (°C)** | 873 | 17.79 | 4.56 | 17.53 | 5.16 | 30.52 |
| **Sun (KJ/m²)** | 873 | 16436.77 | 1639.97 | 16397.88 | 12998.22 | 20942.67 |
| **Male (%)** | 873 | 49.59 | 1.62 | 49.38 | 44.96 | 63.72 |
| **Hispanic (%)** | 873 | 8.83 | 13.40 | 3.31 | 0.36 | 81.84 |
| **Caucasian (%)** | 873 | 86.84 | 15.70 | 93.55 | 3.87 | 99.65 |
| **Poverty (%)** | 873 | 15.32 | 5.36 | 14.58 | 2.98 | 41.68 |
| **Population Density (People per km²)** | 873 | 87.18 | 313.41 | 23.77 | 0.28 | 6437.80 |
| **Current Smoker (%)** | 873 | 25.83 | 4.43 | 26.29 | 9.30 | 39.74 |
| **Obese (%)** | 873 | 24.80 | 4.06 | 25.17 | 12.05 | 37.41 |
|  |  |  |  |  |  |  |
| **Variable** | **N** | **Mean** | **SD** | **Median** | **Minimum** | **Maximum** |
| **Firearm Suicide** | 1173 | 8.51 | 3.82 | 8.09 | 0.77 | 25.60 |
| **Atmospheric Pressure (hPa)** | 1173 | 965.16 | 56.27 | 982.95 | 706.49 | 1014.34 |
| **Weighted Altitude (m)** | 1173 | 431.17 | 533.08 | 258.38 | -9.53 | 3072.03 |
| **Daily Average Max Air Temp (°C)** | 1173 | 17.26 | 4.63 | 16.79 | 6.26 | 30.52 |
| **Sun (KJ/m²)** | 1173 | 16127.71 | 1646.21 | 15978.07 | 12998.22 | 20942.67 |
| **Male (%)** | 1173 | 49.46 | 1.49 | 49.29 | 44.96 | 63.72 |
| **Hispanic (%)** | 1173 | 7.26 | 11.01 | 3.19 | 0.38 | 81.84 |
| **Caucasian (%)** | 1173 | 86.48 | 14.94 | 93.04 | 3.87 | 99.65 |
| **Poverty (%)** | 1173 | 13.81 | 5.19 | 13.22 | 2.98 | 36.01 |
| **Population Density (People per km²)** | 1173 | 184.77 | 1044.89 | 32.32 | 0.28 | 26357.47 |
| **Current Smoker (%)** | 1173 | 25.33 | 4.03 | 25.64 | 9.30 | 36.88 |
| **Obese (%)** | 1173 | 24.71 | 3.73 | 24.97 | 12.05 | 37.05 |
|  |  |  |  |  |  |  |
| **Variable** | **N** | **Mean** | **SD** | **Median** | **Minimum** | **Maximum** |
| **Other Suicide** | 67 | 7.50 | 5.02 | 6.84 | 1.00 | 38.37 |
| **Atmospheric Pressure (hPa)** | 67 | 907.18 | 66.63 | 905.75 | 797.53 | 1014.34 |
| **Weighted Altitude (m)** | 67 | 986.26 | 647.73 | 985.41 | -9.53 | 2077.41 |
| **Daily Average Max Air Temp (°C)** | 67 | 20.06 | 4.95 | 20.97 | 10.37 | 30.52 |
| **Sun (KJ/m²)** | 67 | 18141.15 | 2089.63 | 18993.70 | 14235.45 | 20942.67 |
| **Male (%)** | 67 | 49.72 | 1.25 | 49.47 | 47.69 | 55.36 |
| **Hispanic (%)** | 67 | 24.50 | 23.00 | 17.27 | 0.44 | 81.84 |
| **Caucasian (%)** | 67 | 82.88 | 22.09 | 92.11 | 11.17 | 98.76 |
| **Poverty (%)** | 67 | 18.45 | 6.57 | 17.89 | 8.35 | 41.00 |
| **Population Density (People per km²)** | 67 | 30.21 | 51.23 | 11.90 | 0.87 | 278.03 |
| **Current Smoker (%)** | 67 | 25.49 | 4.26 | 25.45 | 17.33 | 40.29 |
| **Obese (%)** | 67 | 23.96 | 4.20 | 24.11 | 12.26 | 35.87 |
