# Supplementary material for: Modeling the effects of atmospheric pressure on suicide rates in the USA using geographically weighted regression
Source: PLoS One. 2018 Dec 5;13(12):e0206992. doi: 10.1371/journal.pone.0206992 (PMC6281181; doi:10.1371/journal.pone.0206992)
Supplement: S3 Table — (DOCX) [file pone.0206992.s003.docx]

| **Variable** | **N** | **Mean** | **SD** | **Median** | **Minimum** | **Maximum** |
| --- | --- | --- | --- | --- | --- | --- |
| **All Suicide** | 385 | 17.35 | 6.03 | 16.78 | 6.14 | 51.99 |
| **Atmospheric Pressure (hPa)** | 385 | 904.55 | 77.17 | 909.73 | 690.00 | 1014.34 |
| **Weighted Altitude (m)** | 385 | 1003.97 | 746.21 | 926.66 | -9.53 | 3246.61 |
| **Daily Average Max Air Temp (°C)** | 385 | 16.01 | 4.60 | 15.53 | 5.16 | 30.52 |
| **Sun (KJ/m²)** | 385 | 16830.13 | 1942.29 | 16707.52 | 12998.22 | 20942.67 |
| **Male (%)** | 385 | 50.18 | 1.63 | 49.85 | 44.96 | 63.72 |
| **Hispanic (%)** | 385 | 15.59 | 17.30 | 8.91 | 0.36 | 81.84 |
| **Caucasian (%)** | 385 | 91.46 | 11.95 | 94.83 | 3.87 | 99.63 |
| **Poverty (%)** | 385 | 14.83 | 5.18 | 14.07 | 2.98 | 41.68 |
| **Population Density (People per km²)** | 385 | 68.91 | 356.38 | 11.69 | 0.28 | 6437.80 |
| **Current Smoker (%)** | 385 | 23.87 | 4.72 | 23.97 | 9.30 | 39.74 |
| **Obese (%)** | 385 | 22.75 | 4.40 | 22.86 | 12.05 | 37.05 |
|  |  |  |  |  |  |  |
| **Variable** | **N** | **Mean** | **SD** | **Median** | **Minimum** | **Maximum** |
| **Firearm Suicide** | 574 | 9.17 | 4.27 | 8.40 | 1.73 | 25.60 |
| **Atmospheric Pressure (hPa)** | 574 | 940.81 | 69.39 | 970.68 | 706.49 | 1014.34 |
| **Weighted Altitude (m)** | 574 | 658.83 | 663.89 | 362.00 | -9.53 | 3072.03 |
| **Daily Average Max Air Temp (°C)** | 574 | 15.83 | 3.81 | 15.67 | 6.26 | 30.52 |
| **Sun (KJ/m²)** | 574 | 16075.99 | 1848.78 | 15579.60 | 12998.22 | 20942.67 |
| **Male (%)** | 574 | 49.75 | 1.52 | 49.57 | 44.96 | 63.72 |
| **Hispanic (%)** | 574 | 10.10 | 14.00 | 4.46 | 0.38 | 81.84 |
| **Caucasian (%)** | 574 | 90.48 | 11.49 | 94.41 | 3.87 | 99.63 |
| **Poverty (%)** | 574 | 13.25 | 4.75 | 12.90 | 2.98 | 34.75 |
| **Population Density (People per km²)** | 574 | 140.45 | 464.20 | 27.43 | 0.28 | 6437.80 |
| **Current Smoker (%)** | 574 | 24.54 | 4.32 | 24.85 | 9.30 | 36.04 |
| **Obese (%)** | 574 | 23.64 | 3.78 | 24.12 | 12.05 | 37.05 |
|  |  |  |  |  |  |  |
| **Variable** | **N** | **Mean** | **SD** | **Median** | **Minimum** | **Maximum** |
| **Other Suicide** | 5 | 6.15 | 2.99 | 7.13 | 1.00 | 8.42 |
| **Atmospheric Pressure (hPa)** | 5 | 965.88 | 15.48 | 966.86 | 942.21 | 980.24 |
| **Weighted Altitude (m)** | 5 | 406.25 | 142.83 | 393.31 | 276.66 | 627.90 |
| **Daily Average Max Air Temp (°C)** | 5 | 13.77 | 3.64 | 12.33 | 11.21 | 19.98 |
| **Sun (KJ/m²)** | 5 | 15036.98 | 1003.34 | 14616.00 | 14335.64 | 16786.73 |
| **Male (%)** | 5 | 49.44 | 0.58 | 49.28 | 48.86 | 50.30 |
| **Hispanic (%)** | 5 | 2.46 | 1.37 | 3.23 | 0.44 | 3.64 |
| **Caucasian (%)** | 5 | 95.97 | 2.22 | 95.75 | 93.04 | 98.76 |
| **Poverty (%)** | 5 | 12.16 | 4.12 | 11.11 | 9.25 | 19.35 |
| **Population Density (People per km²)** | 5 | 27.51 | 28.69 | 20.03 | 0.87 | 75.26 |
| **Current Smoker (%)** | 5 | 23.48 | 3.45 | 21.94 | 20.77 | 29.28 |
| **Obese (%)** | 5 | 25.19 | 2.09 | 24.34 | 23.27 | 28.61 |

*All significant Bonferroni corrected atmospheric pressure coefficients were negative
